# Antimicrobial Stewardship in Cardiac Device Surgery: Impact of Behavioural Change Interventions on Extended Prophylaxis Practices

**DOI:** 10.3390/antibiotics14080754

**Published:** 2025-07-25

**Authors:** Li Wen Loo, Yvonne Peijun Zhou, Yi Bo Wang, Lai Wei Lee, Jasmine Shimin Chung

**Affiliations:** 1 Division of Pharmacy, Singapore General Hospital, Outram Road, Singapore 169608, Singapore; yvonne.zhou.p.j@sgh.com.sg (Y.P.Z.); wang.yibo@sgh.com.sg (Y.B.W.); lee.lai.wei@sgh.com.sg (L.W.L.); 2 Department of Infectious Diseases, Singapore General Hospital, Outram Road, Singapore 169608, Singapore

**Keywords:** antimicrobial stewardship, antibiotic use, pacemaker insertion, generator change, Cardiology

## Abstract

Background/Objectives: Single-dose pre-operative antibiotic prophylaxis for cardiac-device implantation is recommended but extending antibiotic prophylaxis is common. Locally, 50–60% of patients had extended prophylaxis after pacemaker insertion or generator change. Our antimicrobial stewardship programme (ASP) incorporated behavioural change strategies in implementing a multi-pronged intervention bundle to address this and evaluated its effectiveness and safety. Methods: This single-centre, retrospective cohort study included patients aged 21 years old or older, undergoing uncomplicated pacemaker insertion or generator change at Singapore General Hospital (SGH) from October 2022 to March 2025. To improve antibiotic use, ASP interventions incorporating behaviour change strategies were implemented, namely (1) data-driven feedback, (2) targeted education, (3) identification and engagement of ASP champion, and (4) clinical pathway revision. Results: There were 779 patients evaluated; 380 (48.8%) received standard prophylaxis while 399 (51.2%) received extended prophylaxis with oral antibiotics (mean duration, 3.3 ± 0.8 days). Following ASP interventions, the practice of extended prophylaxis declined significantly from 43.8% to 24.0% (*p* < 0.01). The incidence of surgical site infections was low and similar in both groups (0.8%, *p* = 1.000); all infections were superficial. There was also significant reduction in the proportion of patients on all antibiotics from 20.7% to 16.3% (*p* < 0.01). Identification and engagement of ASP champion proved pivotal in changing prescribing behaviour through peer influence and credibility. Conclusions: The bundled ASP interventions, incorporating behavioural change strategies, have effectively and safely reduced the use of extended prophylaxis post-cardiac device implantation. Behavioural change interventions are essential to achieve sustained stewardship success.

## 1. Introduction

The antimicrobial stewardship programme (ASP) in healthcare institutions is advocated worldwide to curb rising antimicrobial resistance rates [1]. At Singapore General Hospital (SGH), an official ASP was established in 2008, comprising infectious diseases physicians and pharmacists. Since then, we have implemented various antimicrobial stewardship strategies, including prospective audit and feedback (PAF), formulary restrictions, antibiotic guideline development, and Computerised Decision Support System (CDSS) to improve the appropriate use of broad-spectrum intravenous (IV) antibiotics (carbapenems, piperacillin-tazobactam, ceftriaxone, and IV fluoroquinolones). These ASP interventions have been shown to be safe and associated with a significant reduction in the incidence density of antibiotic-resistant organisms, length of hospitalisation, and healthcare cost [2,3,4].

However, there remains room for improvement in antibiotic use. Overall antibiotic consumption remains high; approximately 50% of patients admitted to public hospital in Singapore were prescribed at least one antibiotic [5]. A 2022-point prevalence survey (PPS) conducted at SGH suggested that it was imperative for SGH–ASP to expand our purview beyond broad-spectrum IV antibiotics; 46.3% of all our inpatients were on antibiotics, a higher prevalence than Europe (31.9%) and North America (38.6%) [6]. Notably, oral antibiotics represent 36.9% of all antibiotics prescribed and specifically, its use in extended post-surgery surgical prophylaxis remains a concern in our institution.

Among inpatients who underwent surgical procedures, our in-house PPS also revealed that there was high prevalence (58.6%) of patients receiving more than 24 h of surgical prophylaxis post procedure. A deep dive into surgical prophylaxis uses across various departments showed that 43.8% of the patients who underwent pacemaker insertions or generator change were prescribed oral antibiotics as extended surgical prophylaxis post procedure in October 2022. Furthermore, these oral antibiotics contributed to about 15% of the total antibiotic consumption in the Department of Cardiology, which may have driven the overuse of antibiotics.

Both the American Heart Association and European Society of Cardiology recommend a single pre-operative dose of cefazolin (or vancomycin for β-lactam allergies) for uncomplicated cardiac implantable electronic device (CIED) surgeries [7,8]. This has been shown to reduce the risk of infection, and is effective in reducing morbidity, prolonged hospitalisation, and healthcare costs. However, there is a lack of evidence to support the continued use of prophylactic antibiotics post-implantation of pacemaker or generator change [9,10,11,12,13,14,15].

Despite the paucity of evidence, prescribing oral antibiotics post-pacemaker insertion or generator change remains prevalent in our hospital. Typically, patients receive standard intravenous (IV) cefazolin (or vancomycin for patients with β-lactam allergy or MRSA colonised), followed by 3 to 5 days of oral antibiotics post procedure. This extended surgical prophylaxis use was largely driven by legacy beliefs that antibiotics reduce the risk of surgical site infections (SSIs). These beliefs became embedded into a legacy coordinated clinical pathway (CCP) for pacemaker insertion and generator change in the electronic health records, which facilitated easy prescription of antibiotics post surgery.

In January 2023, the ASP team implemented a multi-pronged strategy to drive behavioural change amongst cardiologists, with the goal to promote appropriate antibiotic use in surgical prophylaxis. This strategy included (1) the implementation of a data-driven feedback system through report dissemination to the Head of Department; (2) stakeholder engagement by identifying ASP champions within the department and partnering them in ASP activities; (3) targeted education sessions for senior cardiologists; and (4) clinical pathway optimisation.

The primary outcome of our study is to evaluate the impact of the multi-pronged ASP strategy in reducing the proportion of patients who were prescribed oral antibiotics as extended surgical prophylaxis post-pacemaker insertion or generator change. The secondary outcomes include (1) comparing the surgical site infection (SSI) rates of patients who received standard surgical prophylaxis and extended surgical prophylaxis with oral antibiotics; and (2) evaluating the proportion of inpatients on any antibiotics for all indications (not limited to surgical prophylaxis use) in the Department of Cardiology within the same study period.

## 2. Methodology

### 2.1. Study Design and Setting

This is a single-centre, retrospective study conducted at SGH, the largest acute-tertiary care hospital in Singapore, with a capacity of 2000 beds. For the evaluation of antibiotic use in surgical prophylaxis and SSI, all patients aged 21 years and above, admitted under the Department of Cardiology for pacemaker insertion or generator change from October 2022 to March 2025 were included in the study. Patients who had a complicated procedure (as defined by prolonged procedure, massive bleeding and post-operative haematoma) or had concurrent infections were excluded.

To calculate the proportion of inpatients who received antibiotics, we included all patients who were admitted under the Department of Cardiology at SGH from October 2022 to March 2025. The monthly proportion was derived by dividing the total number of days where patients were prescribed at least one antibiotic (for any indication) by the total number of inpatient days in the Department of Cardiology. The KPI for proportion of patients receiving all antibiotics was calculated by dividing the number of inpatient days with antibiotics given by the total number of inpatient days in the Department of Cardiology.

### 2.2. Data Collection

All data were collected using the hospital electronic health records. Patient demographics collected included age, gender, body weight, medical comorbidities and *Methicillin-resistant Staphylococcus aureus* (MRSA) colonisation status. Pertaining to surgical prophylaxis, the choice, route and, duration of antibiotics were collected.

Incidence of SSI (up to 30 days post-pacemaker insertion or generator change) was also collected. SSI is classified as (i) superficial incisional SS; (ii) deep incisional SSI; or (iii) Organ/Space SSI, based on the definitions from the Centres for Disease Control (CDC)/National Healthcare Safety Network (NHSN) [16]. SSI diagnosis was confirmed based on clinical documentation in our hospital’s electronic patient records.

In addition, information on the monthly proportion of patients on antibiotics in the Department of Cardiology was also extracted from the hospital data warehouse (Electronic Health Intelligence System).

### 2.3. ASP Interventions

Our multi-pronged strategy includes the following:

#### 2.3.1. Implementation of a Data-Driven Feedback System Specifically on Antibiotic Use in Surgical Prophylaxis

Since the implementation of ASP, we have been disseminating quarterly reports to all the Heads of Department of Cardiology. These reports typically describe the department’s antibiotic prescribing performance, including the appropriateness of antibiotics that were audited by ASP pharmacists through PAF. However, the purview of ASP had expanded beyond the use of IV broad spectrum. From January 2023, analysis on the number of patients who received oral antibiotics as extended surgical prophylaxis post-pacemaker insertion or generator change was included. Additionally, these analyses were shared at department meetings in August 2023 and October 2024.

#### 2.3.2. Targeted Education Sessions for Cardiologists

Following the dissemination of reports, we recognised the importance of driving behavioural change through interactive education sessions with the cardiologists. Strong legacy beliefs on the use of extended antibiotics to reduce risk of SSI were deeply entrenched amongst the cardiologists, and it was pertinent for ASP to debunk the myths with evidence-based data. Targeted education sessions were conducted in August 2023 and October 2024, where the literature review pertaining to surgical prophylaxis for CIED implantation and associated outcomes including SSI were shared. The cardiologists’ concerns were acknowledged, and the ASP team had engaged them in in-depth discussions on how to improve antibiotic use.

#### 2.3.3. Stakeholder Engagement by Identifying ASP Champions in Department of Cardiology and Partnering Them in ASP Activities

Apart from “big group” education sessions, we recognised the importance of engaging physicians individually. Hence, in January 2024, ASP identified a senior cardiologist as our ASP champion for the Department of Cardiology. He played a key role in promoting stewardship efforts within the department, encouraging appropriate antibiotic prescribing habits through peer influence, encouragement, empowerment, and education.

#### 2.3.4. Clinical Pathway Optimisation Through Revisions to the Coordinated Clinical Pathway (CCP) for Pacemaker Insertion and Generator Change

During the engagement sessions with the senior cardiologists, we identified that oral antibiotics intended for extended surgical prophylaxis was included as part of the department CCP for post-pacemaker insertion and generator change. Hence, physicians who selected this CCP in the patient’s electronic record peri-procedure, will have oral antibiotics prescribed for the patients’ post procedure. After extensive discussions with the cardiologists, the use of post-procedural oral antibiotics in the CCPs was removed in June 2024.

### 2.4. Statistical Methods

The IBM SPSS Statistics (Version 26.0, IBM Corp, Armonk, NY, USA) was used for statistical calculations. For categorical variables, data was analysed using χ^2^ test or Fisher’s exact test, as appropriate. Linear regression was performed to evaluate the impact of our multi-pronged ASP strategy in reducing the proportion of patients who received extended surgical prophylaxis with oral antibiotics, and the proportion of cardiology in patients who received antibiotics (for all indications) with time.

## 3. Results

Between October 2022 to March 2025, a total of 818 patients were admitted to the Department of Cardiology for pacemaker insertions or generator change. Out of the 818 cases, 4.8% (39/818) were excluded as they had complicated procedures, and 779 patients were included in our study [Table 1].

### 3.1. Surgical Prophylaxis

The choice of surgical prophylaxis was in accordance with our hospital guidelines—intravenous cefazolin or vancomycin for patients with β-lactam allergy or MRSA colonisation. Of the 779 patients included in our study, 48.8% (380/779) received standard surgical prophylaxis while 51.2% (399/779) received extended surgical prophylaxis with oral antibiotics post-pacemaker insertion or generator change.

For patients who were given standard prophylaxis, the majority were given IV Cefazolin (304/380, 80.0%), while the remaining were given IV Vancomycin (76/380, 20.0%). Amongst those who received extended surgical prophylaxis with oral antibiotics, 368/399 (92.2%) and 8/399 (2.0%) received oral cefuroxime and cefalexin, respectively. For the remaining 23/399 (5.8%) patients with β-lactam allergy, oral clindamycin was prescribed. The median duration of extended oral antibiotics was 3.3 ± 0.8 days [Table 1].

**Figure 1:**
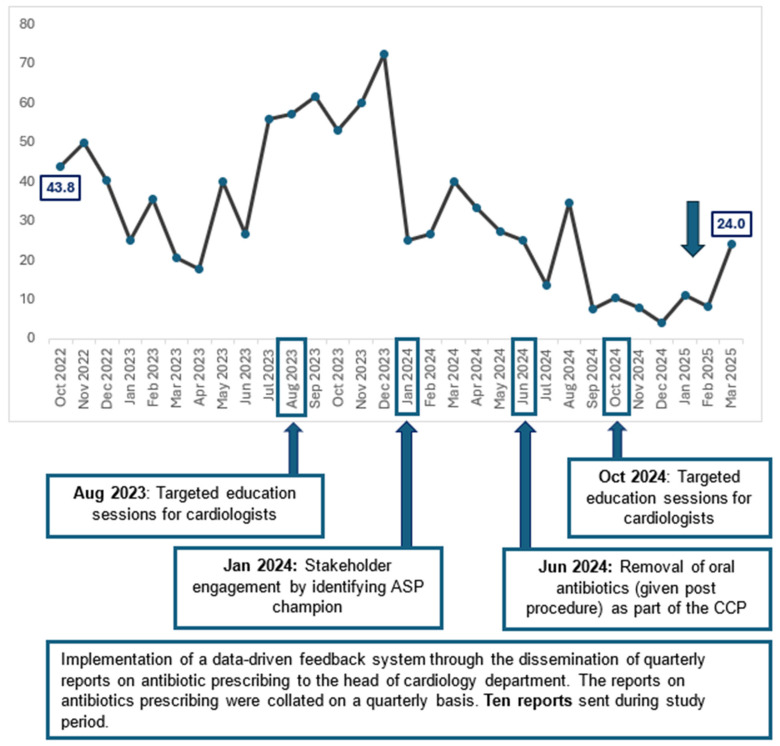
Percentage of patients with extended surgical prophylaxis post-pacemaker insertion or generator change (October 2022 to March 2025).

When trended over time, there was a significant reduction in the proportion of patients who were prescribed oral antibiotics as extended surgical prophylaxis post procedure (*p* < 0.01) from 43.8% in October 2022 to 24.0% in March 2025 [Figure 1].

### 3.2. Surgical Site Infection Rates

There was no significant difference in SSI incidence in the standard (3/380, 0.8%) and extended surgical prophylaxis (3/399, 0.8%) group (*p* = 1.0). All six patients in both groups had superficial incisional SSI. None were colonised with MRSA, and all were treated with a 5-day course of oral co-amoxiclav and discharged well.

### 3.3. Proportion of Inpatients on Antibiotics

**Figure 2:**
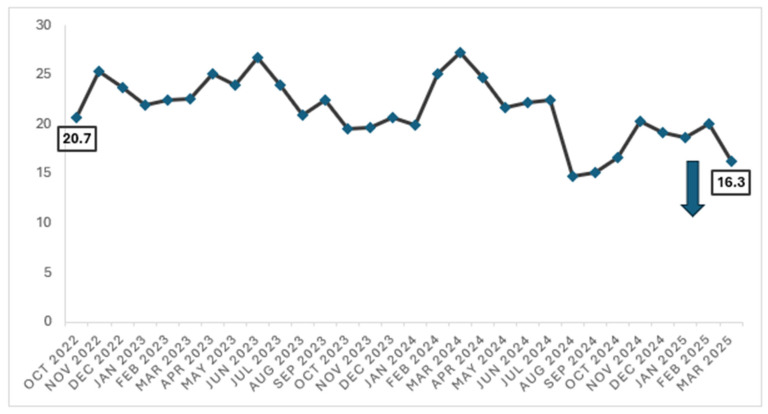
Proportion of patients on any antibiotics (for all indications) in the Department of Cardiology (October 2022 to March 2025).

When trend over time, there was a significant reduction in the proportion of cardiology inpatients who were prescribed antibiotics (for any indication) from 20.7% in October 2022 to 16.3% in March 2025 (*p* < 0.01) [Figure 2].

## 4. Discussion

Conventionally, our local stewardship efforts were focused on reducing inappropriate use of IV broad-spectrum antibiotics (e.g., carbapenems, piperacillin-tazobactam, ceftriaxone, intravenous fluoroquinolones). While appropriate and impactful initially, restricting stewardship to only broad-spectrum antibiotics represents a narrow approach towards antimicrobial stewardship. Since the inception of the ASP programme, the appropriateness of IV broad-spectrum antibiotics has consistently exceeded 80%. A broader approach would involve expanding the purview of audit to include a targeted audit of narrow spectrum antibiotics with potential for overuse, and review of surgical prophylaxis fits this indication. Based on PPS conducted at our institution, improving surgical prophylaxis in the cardiology department was an area we can effect a positive change.

The literature surrounding ASP strategies targeted at cardiology interventions is limited. A paper by Surat et al. demonstrated that structured antimicrobial stewardship interventions can optimise peri-operative antibiotic use without compromising patient safety in cardiothoracic surgery [17]. Our study is the first to describe the impact of antimicrobial stewardship on antibiotic use in pacemaker insertion or generator change.

**Figure 3:**
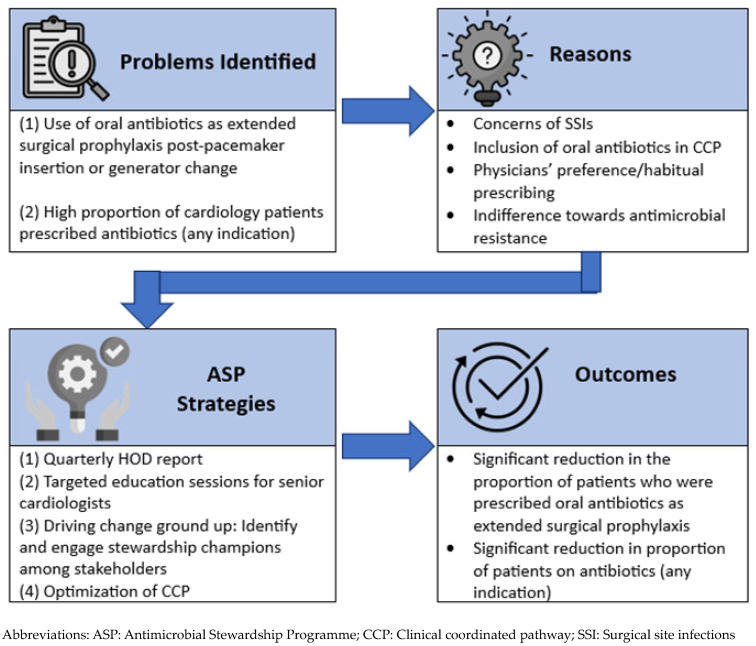
Our ASP approach to reducing antibiotic prescription.

We adopted a systematic approach to address the issue of extended surgical prophylaxis by first identifying the underlying reasons for inappropriate prescribing, followed by implementing targeted strategies for each contributing factor [Figure 3]. The incorporation and application of behavioural sciences, supported by multi-disciplinary collaboration, underpinned our approach to reduce unnecessary antibiotics use post procedurally [18,19]. In 2014, the National Institute of Health and Care Excellence (NICE) identified several evidence-based techniques that are relevant to changing professional behaviour in clinical settings. The four core elements of the behavioural change techniques identified are—goal setting, self-monitoring, feedback, and action planning [19].

Research has shown that setting specific and measurable goals, especially when coupled with feedback—is a powerful stimulus for behavioural change [19]. For goal setting at SGH, since 2020, we have individualised and set targets for an overall proportion of patients on antibiotics for each department based on departmental case mix and trends with time. However, antibiotic use was consistently higher than the projected target in the Department of Cardiology. Based on PPS performed in 2022, the ASP identified the overuse of antibiotic prophylaxis as a target for stewardship intervention. Through targeted education sessions—we encouraged physicians to review their antibiotic prophylaxis use, and provided summaries of latest evidence-based recommendations in order equip them with the relevant knowledge to limit antibiotic prescription post uncomplicated cardiac procedures. To follow up on these education sessions, regular and timely feedback was provided through quarterly reports on antibiotics use and prescribing patterns of ASP-audited antibiotics. These reports were disseminated through the Head of Department of Cardiology and shared during department-wide engagement sessions. This is based on the premise that enabling interventions with feedback were more effective than those without [20]. When we observed that this ‘large-scale feedback’ alone at the cardiology departmental platforms was inadequate to drive significant behavioural change, we then identified and engaged the support of a highly esteemed senior cardiologist as our ASP champion. Together with other relevant stakeholders, we worked to promote stewardship efforts within the department, including the removal of oral antibiotics as extended surgical prophylaxis in the CCP.

### 4.1. Impact of ASP on Extended Surgical Prophylaxis

Over the two-and-a-half-year study period, our multi-pronged ASP strategy significantly reduced the proportion of patients who were prescribed oral antibiotics as extended surgical prophylaxis post-pacemaker insertion or generator change (*p* = 0.002). These interventions, aimed at promoting adherence to standard antibiotic prophylaxis protocol, were found to be safe. The SSI rates remained low at 0.8% in both groups, comparable to SSI rates in the published literature (ranging from 0.63% to 3.28%) [5,6,7].

While the combined ASP strategies were effective overall, we acknowledge that individual strategies may be less effective on their own. Initially, we believed that quarterly HOD reports would suffice as an effective feedback mechanism. However, the proportion of patients receiving extended prophylaxis with oral antibiotics fluctuated, showing no consistent downward trend. The lack of efficacy of this approach may be attributed due to manpower fluctuations, the failure of information to reach all the treating physicians (including junior doctors that rotated through the cardiology department), or that some cardiologists may have received the report but did not pay attention to the contents.

Henceforth, we initiated a targeted education session for cardiologists in August 2023, where published literature on the safety of limiting surgical prophylaxis to pre-surgery was shared. While some studies reported that educational interventions significantly reduced overall antibiotic prescription rates, this strategy did not appear effective for us [21]. Ironically, we saw a slight increase in percentage of patients on extended prophylaxis with oral antibiotics from 57.1% (August 2023) to 61.5% (September 2023). In the post-COVID-19 era, many meetings were still conducted virtually, as were the education sessions that we conducted. This might be a possible reason for the lack of efficacy of our education sessions due to the challenges of engaging most of the audience on a virtual platform.

In our study, we observed that stakeholder engagement by identifying ASP champions in the Department of Cardiology and partnering them in ASP activities appears to be the most impactful [Figure 1]. In January 2024, we identified a prominent senior cardiologist as our ASP champion, who actively engaged other cardiologists to drive behavioural change through (i) presence of social proof and peer influence—clinicians are more likely to change when feedback comes from a peer, (ii) credibility and trust—ASP champions bring clinical relevance to our stewardship recommendations, and (iii) visibility and reinforcement [22,23]. Antibiotic champions have also been quoted as one of the most effective interventions to optimise antibiotic prescribing in focus group interviews conducted by Borek et al. [24]. Expectedly, following the ASP champion engagement, we observed a sharp drop in the percentage of patients on extended prophylaxis with oral antibiotics from 72.4% in December 2023 to 25.0% in January 2024.

Understanding workflow processes within the Department of Cardiology was also important. It was through the engagement of the ASP champion that we were made aware of the integration of oral antibiotics into the department’s CCP for pacemaker and generator change procedure. The removal of oral antibiotic use post procedure from the CCP had the second-most prominent and sustainable impact on extended surgical prophylaxis.

In September 2024, we achieved and sustained having less than 20% of patients prescribed with extended surgical prophylaxis. Notably, we observed a spike in percentage of patients on extended prophylaxis with oral antibiotics in March 2025, which was contributed by a group of new visiting consultants. Due to schedules of the visiting consultants, they may not always be available for the scheduled stewardship interventions with the rest of the cardiology department. This was a reminder for the stewardship to engage the visiting consultants in our subsequent stewardship outreach, and this may involve a small group session which may best fit their schedules, or an individualised antibiotic prescribing recommendations could be shared. As old habits die hard, we acknowledge that our study period of two and a half year may not be adequate to obliterate all inappropriate antibiotic prescribing habits. Continued handshake stewardship is pertinent in optimising antibiotic prescribing [25,26,27].

### 4.2. Impact of Standard vs. Extended Prophylaxis on Surgical Site Infections, Clinical Care and Healthcare Costs

There was no significant difference in SSI incidence in the standard (3/380, 0.8%) and extended surgical prophylaxis (3/399, 0.8%) group (*p* = 1.0). While cost savings attributed to drug cost alone because of reducing antibiotic use post surgery is not astronomical (approximately SGD 5 dollars per patient for a 3-day course of oral antibiotics), there is an expected reduction in healthcare cost saving due to the reduced selection pressure for antibiotic-resistant organisms and *Clostridioides difficile* infection [28]. Notably, extended use of antibiotics post-cardiac device procedures have shown to increase the risk of *C. difficile*-associated diarrhoea and incidence of acute kidney injury [29]. Additionally, we observed that some of the patients in our study cohort were also prescribed warfarin, an anticoagulant. Patients who are on concurrent antibiotics and warfarin have an increased risk of bleeding due to drug–drug interactions [30,31,32] and may necessitate more frequent checks to trend their coagulation profile, adding to the rising healthcare cost for both patients and our healthcare system.

### 4.3. Impact of ASP Interventions on Surgical Prophylaxis with Spillover Effects on Antibiotic Consumption

Interestingly, we have also observed a significant reduction in the proportion of patients who were prescribed any antibiotic (for all indications) from 20.7% in October 2022 to 16.3% in March 2025 (*p* < 0.01). This reduction translates to approximately 1700 antibiotic-free days in a year, based on a monthly average of 3200 patient days for the Department of Cardiology. The overall reduction in antibiotic use within the department strongly suggests that, while our main ASP intervention targeted inappropriate antibiotic use in surgical prophylaxis, the constant presence of ASP, coupled with the multi-pronged stewardship efforts, have encouraged cardiologists to be more cognisant in prescribing antibiotics appropriately. Notably, the target of reducing the proportion of inpatients prescribed with antibiotics to less than 18% was achieved in August 2024 till October 2024. While there was a slight increase in the proportion thereafter, an improvement was observed again in March 2025, reiterating the importance of the ongoing engagement of clinicians.

### 4.4. Study Limitations

Our study has several limitations. Firstly, we excluded patients who had complications arising from surgery (i.e., prolonged procedure, massive bleeding, and post-operative haematoma), thus limiting generalisability to high-risk populations. Future prospective studies can be conducted to address this gap. Secondly, our study’s retrospective nature may introduce confounders. We acknowledge that patient outcomes are not evaluated prospectively, and our analysis is limited by available clinical information in our hospital electronic records, which may be incomplete. For example, patients may have attended another healthcare facility to evaluate for potential SSIs after discharge. However, as SGH is the largest tertiary healthcare institution in Singapore and patients are generally counselled to return to us for potential medical issues, the likelihood of missing out on follow-ups is low. Thirdly, this is a single-centre study and selected stewardship strategies may not be generalisable to other settings that may differ in hierarchical structure, local prescribing culture, and hospital infrastructure. Nevertheless, our study highlights the importance of identifying and collaborating with like-minded colleagues who have an interest in improving antimicrobial prescribing. Additionally, our study proved that incorporating appropriate antibiotic-related recommendations in a surgical coordinated clinical pathway, which could be accessible electronically (or in hardcopy format), is essential in advocating adherence to best practices in patient care. Lastly, a longer study period may be needed to show the effects of a sustainable stewardship strategy on antibiotic prescribing.

## 5. Conclusions

In this study, we showed that our structured and multi-pronged stewardship strategy were effective in reducing the proportion of inpatients on extended surgical prophylaxis and inpatients on antibiotics for all indications. The introduction of a respected physician as our ASP Champion was found to be the key to our success. Effective engagement of clinicians was also crucial in improving antibiotic prescribing. Additionally, we demonstrated that short-course surgical prophylaxis was safe and did not compromise patient outcomes. It is important for antimicrobial stewardship programmes to remain insightful and adaptable in driving targeted ASP strategies to promote cognisant prescribing amongst clinicians.

## Figures and Tables

**Table 1:** Patient demographics.

	Standard Prophylaxis N = 380	Extended Prophylaxis N = 399	*p*-Value
Male (%)	241 (63.4)	244 (61.2)	0.514
Patients with diabetes mellitus (%)	131 (34.5)	147 (36.8)	0.446
Mean weight (kg) ± S.D.	62.7 ± 15.9	62.4 ± 14.2	0.295
Mean age ± S.D.	72.5 ± 12.3	73.0 ± 13.9	0.728
No. of MRSA colonisation (%)	6 (1.6)	9 (2.3)	0.492
Antibiotics used in prophylaxis			
IV Cefazolin (%)	304 (80.0)	N.A.	
IV Vancomycin (%)	76 (20.0)	N.A.	
PO Cefalexin (%)	N.A.	8 (2.0)	
PO Cefuroxime (%)	N.A.	368 (92.2)	
PO Clindamycin (%)	N.A.	23 (5.8)	
Mean duration of oral antibiotics used in extended prophylaxis ± S.D. (days)	N.A.	3.3 ± 0.8	
No. of surgical site infections (%)	3 (0.8)	3 (0.8)	

Abbreviations: IV: Intravenous; MRSA: *Methicillin-resistant Staphylococcus aureus*; N.A.: Not applicable; PO: Per oral; S.D: Standard deviation.

## Data Availability

Restrictions apply to the datasets. The datasets presented in this article are not readily available as they are part of an ongoing study. Requests to access the datasets should be directed to Li Wen Loo (loo.li.wen@sgh.com.sg).

## References

[1] Barlam T.F., Cosgrove S.E., Abbo L.M., MacDougall C., Schuetz A.N., Septimus E.J., Srinivasan A., Dellit T.H., Falck-Ytter Y.T., Fishman N.O. (2016). SHEA/IDSA Clinical Practice Guidelines for Implementing an Antimicrobial Stewardship Program. Clin. Infect. Dis..

[2] Loo L.W., Liew Y.X., Lee W., Lee L.W., Chlebicki P., Kwa A.L.-H. (2019). Discontinuation of antibiotic therapy within 24 hours of treatment initiation for patients with no clinical evidence of bacterial infection: A 5-year safety and outcome study from Singapore General Hospital Antimicrobial Stewardship Program. Int. J. Antimicrob. Agents.

[3] Liew Y.X., Lee W., Loh J.C.Z., Cai Y., Tang S.S.L., Lim C.L.L., Teo J., Ong R.W.Q., Kwa A.L.-H., Chlebicki M.P. (2012). Impact of an antimicrobial stewardship programme on patient safety in Singapore General Hospital. Int. J. Antimicrob. Agents.

[4] Ng T.M., Ang L.W., Heng S.T., Kwa A.L.-H., Wu J.E., Seah X.F.V., Lee S.Y., Seah J., Choo R., Lim P.L. (2023). Antibiotic utilisation and resistance over the first decade of nationally funded antimicrobial stewardship programmes in Singapore acute-care hospitals. Antimicrob. Resist. Infect. Control.

[5] Cai Y., Venkatachalam I., Tee N.W., Tan T.Y., Kurup A., Wong S.Y., Low C.Y., Wang Y., Lee W., Liew Y.X. (2017). Prevalence of Healthcare-Associated Infections and Antimicrobial Use Among Adult Inpatients in Singapore Acute-Care Hospitals: Results From the First National Point Prevalence Study. Clin. Infect. Dis..

[6] Versporten A., Zarb P., Caniaux I., Gros M.-F., Drapier N., Miller M., Jarlier V., Nathwani D., Goossens H., Koraqi A. (2018). Antimicrobial consumption and resistance in adult hospital inpatients in 53 countries: Results of an internet-based global point prevalence study. Lancet Glob. Health.

[7] Kusumoto F.M., Schoenfeld M.H., Wilkoff B.L., Berul C.I., Birgersdotter-Green U.M., Carrillo R., Cha Y.-M., Clancy J., Deharo J.-C., Ellenbogen K.A. (2017). 2017 HRS expert consensus statement on cardiovascular implantable electronic device lead management and extraction. Heart Rhythm.

[8] Baddour L.M., Epstein A.E., Erickson C.C., Knight B.P., Levison M.E., Lockhart P.B., Masoudi F.A., Okum E.J., Wilson W.R., Beerman L.B. (2010). Update on cardiovascular implantable electronic device infections and their management: A scientific statement from the American Heart Association. Circulation.

[9] Khan N.K., Subramaniam V., Hee C. (2010). Antibiotic prophylaxis for permanent pacemaker implantation: An observational study of practice in England. Br. J. Cardiol.

[10] de Oliveira J.C., Martinelli M., Nishioka S.A.D.O., Varejão T., Uipe D., Pedrosa A.A.A., Costa R., Danik S.B. (2009). Efficacy of antibiotic prophylaxis before the implantation of pacemakers and cardioverter-defibrillators: Results of a large, prospective, randomized, double-blinded, placebo-controlled trial. Circ. Arrhythmia Electrophysiol..

[11] Da Costa A., Kirkorian G., Cucherat M., Delahaye F., Chevalier P., Cerisier A., Isaaz K., Touboul P. (1998). Antibiotic prophylaxis for permanent pacemaker insertion: A meta-analysis. Circulation.

[12] Lee W., Huang T., Lin L., Lee P., Lin C., Lee C., Chao T., Li Y., Chen J. (2017). Efficacy of postoperative prophylactic antibiotics in reducing permanent pacemaker infections. Clin. Cardiol..

[13] Kabulski G.M., Northup A., Wiggins B.S. (2019). Postoperative Antibiotic Prophylaxis Following Cardiac Implantable Electronic Device Placement. J. Innov. Card. Rhythm. Manag..

[14] Krahn A.D., Longtin Y., Philippon F., Birnie D.H., Manlucu J., Angaran P., Rinne C., Coutu B., Low R.A., Essebag V. (2018). Prevention of Arrhythmia Device Infection Trial—The PADIT Trial. J. Am. Coll. Cardiol..

[15] Rennert-May E., Leal J., Zhang Z., Rajakumar I., Smith S., Conly J.M., Exner D., Kuriachan V., Chew D. (2024). Rates of post procedural prophylactic antibiotic use following cardiac implantable electronic device insertion and the impact on surgical site infections in Alberta, Canada. Antimicrob. Resist. Infect. Control.

[16] Centers for Disease Control and Prevention (CDC) (2024). National Healthcare Safety Network (NHSN) Patient Safety Component Manual.

[17] Surat G., Bernsen D., Schimmer C. (2021). Antimicrobial Stewardship measures in cardiac surgery and its impact on surgical site infections. J. Cardiothorac. Surg..

[18] Charani E., Edwards R., Sevdalis N., Alexandrou B., Sibley E., Mullett D., Franklin B.D., Holmes A. (2011). Behavior Change Strategies to Influence Antimicrobial Prescribing in Acute Care: A Systematic Review. Clin. Infect. Dis..

[19] Davey P., Peden C., Charani E., Marwick C., Michie S. (2015). Time for action—Improving the design and reporting of behaviour change interventions for antimicrobial stewardship in hospitals: Early findings from a systematic review. Int. J. Antimicrob. Agents.

[20] Davey P., Marwick C.A., Scott C.L., Charani E., McNeil K., Brown E., Gould I.M., Ramsay C.R., Michie S. (2017). Interventions to improve antibiotic prescribing practices for hospital inpatients (Review). Cochrane Database Syst. Rev..

[21] Zheng K., Xie Y., Dan L., Mao M., Chen J., Li R., Wang X., Hesketh T. (2022). Effectiveness of Educational Interventions for Health Workers on Antibiotic Prescribing in Outpatient settings in China: A Systematic Review and Meta-Analysis. Antibiotics.

[22] Yong C.W., Choe R., Chua S.K.X., Lum J.L., Wang W.C.-W. (2025). Utilising a COM-B framework to modify antibiotic prescription behaviours following third molar surgeries. Ann. Acad. Med. Singap..

[23] Cialdini R.B. (2009). The Psychology of Persuasion (Revised Edition).

[24] Borek A.J., Campbell A., Dent E., Moore M., Butler C.C., Holmes A., McLeod M., Tonkin-Crine S., Anyanwu P.E., on behalf of the STEP-UP study team (2021). Development of an intervention to support the implementation of evidence-based strategies for optimising antibiotic prescribing in general practice. Implement. Sci. Commun..

[25] Hurst A.L., Child J., Pearce K., Palmer C., Todd J.K., Parker S.K. (2016). Handshake Stewardship: A Highly Effective Rounding-based Antimicrobial Optimization Service. Pediatr. Infect. Dis. J..

[26] Neuner E.A., Atkinson A., Ilges D., Krekel T., Ritchie D.J., Bewley A.F., Durkin M.J., Hsueh K., Sayood S. (2023). Mixed methods evaluation of handshake antimicrobial stewardship on adult inpatient medicine floors. Antimicrob. Steward. Healthc. Epidemiol..

[27] Kosharek A., Neuner E., Welch E., January S., Bewley A., Hsueh K., Sayood S. (2025). Handshake Antimicrobial stewardship for adult surgical patients. Antimicrob. Steward. Healthc. Epidemiol..

[28] Martinez-Sobalvarro J.V., Júnior A.A.P., Pereira L.B., Baldoni A.O., Ceron C.S., dos Reis T.M. (2022). Antimicrobial stewardship for surgical antibiotic prophylaxis and surgical site infections: A systematic review. Int. J. Clin. Pharm..

[29] Asundi A., Stanislawski M., Mehta P., Barón A.E., Gold H., Mull H., Ho P.M., Gupta K., Branch-Elliman W. (2018). Prolonged antimicrobial prophylaxis following cardiac device procedures increases preventable harm: Insights from the VA CART program. Infect. Control Hosp. Epidemiol..

[30] Baillargeon J., Holmes H.M., Lin Y.-L., Raji M.A., Sharma G., Kuo Y.-F. (2012). Concurrent Use of Warfarin and Antibiotics and the Risk of Bleeding in Older Adults. Am. J. Med..

[31] Spanakis M., Alon-Ellenbogen D., Ioannou P., Spernovasilis N. (2023). Antibiotics and Lipid-Modifying Agents: Potential Drug-Drug Interactions and Their Clinical Implications. Pharmacy.

[32] Vega A.J., Smith C., Matejowsky H.G., Thornhill K.J., Borne G.E., Mosieri C.N., Shekoohi S., Cornett E.M., Kaye A.D. (2023). Warfarin and Antibiotics: Drug Interactions and Clinical Considerations. Life.

